# Harmonization of quality metrics and power calculation in multi-omic studies

**DOI:** 10.1038/s41467-020-16937-8

**Published:** 2020-06-18

**Authors:** Sonia Tarazona, Leandro Balzano-Nogueira, David Gómez-Cabrero, Andreas Schmidt, Axel Imhof, Thomas Hankemeier, Jesper Tegnér, Johan A. Westerhuis, Ana Conesa

**Affiliations:** 1 Department of Applied Statistics, Operations Research and Quality, Universitat Politècnica de València, Valencia, Spain; 2 Microbiology and Cell Science Department, Institute for Food and Agricultural Research, University of Florida, Gainesville, FL USA; 3 Unit of Computational Medicine, Department of Medicine, Solna, Center for Molecular Medicine, Karolinska Institutet, Stockholm, Sweden; 4 Science for Life Laboratory, Solna, Sweden; 5 Mucosal & Salivary Biology Division, King’s College London Dental Institute, London, UK; 6 Navarrabiomed, Complejo Hospitalario de Navarra (CHN), Universidad Pública de Navarra (UPNA), IdiSNA, Pamplona, Spain; 7 Protein Analysis Unit, Biomedical Center, Faculty of Medicine, LMU Munich, Planegg-Martinsried, Germany; 8 Munich Center of Integrated Protein Science LMU Munich, Planegg-Martinsried, Germany; 9 Division Analytical Biosciences, Leiden/Amsterdam Center for Drug Research, Leiden, The Netherlands; 10 Biological and Environmental Sciences and Engineering Division, Computer, Electrical and Mathematical Sciences and Engineering Division, King Abdullah University of Science and Technology, Thuwal, Saudi Arabia; 11 Swammerdam Institute for Life Sciences, University of Amsterdam, Amsterdam, The Netherlands; 12 Department of Statistics, Faculty of Natural Sciences, North-West University (Potchefstroom Campus), Potchefstroom, South Africa; 13 Genetics Institute, University of Florida, Gainesville, FL USA

**Keywords:** Data integration, Quality control

## Abstract

Multi-omic studies combine measurements at different molecular levels to build comprehensive models of cellular systems. The success of a multi-omic data analysis strategy depends largely on the adoption of adequate experimental designs, and on the quality of the measurements provided by the different omic platforms. However, the field lacks a comparative description of performance parameters across omic technologies and a formulation for experimental design in multi-omic data scenarios. Here, we propose a set of harmonized Figures of Merit (FoM) as quality descriptors applicable to different omic data types. Employing this information, we formulate the MultiPower method to estimate and assess the optimal sample size in a multi-omics experiment. MultiPower supports different experimental settings, data types and sample sizes, and includes graphical for experimental design decision-making. MultiPower is complemented with MultiML, an algorithm to estimate sample size for machine learning classification problems based on multi-omic data.

## Abstract (web summary)

Multi-omics studies are popular but lack rigorous criteria for experimental design. We define Figures of Merit across omics to comparatively describe their performance, and present new algorithms for sample size calculation in multi-omics experiments aiming either at feature selection or sample classification.

## Introduction

The genomics research community has been increasingly proposing the parallel measurement of diverse molecular layers profiled by different omic assays as a strategy to obtain comprehensive insights into biological systems^1–4^. Encouraged by constant cost reduction, data-sharing initiatives, and availability of data (pre)processing methods^5–13^, the so-called multi-platform or multi-omics studies are becoming popular. However, the success of a multi-omic project in revealing complex molecular interconnections strongly depends on the quality of the omic measurement and on the synergy between a carefully designed experimental setup and a suitable data integration strategy. For example, multi-omic measurements should derive from the same samples, observations should be many, and variance distributions similar if the planned approach for data integration relies on correlation networks. Frequently, these issues are overlooked, and analysis expectations are frustrated by underpowered experimental design, noisy measurements, and the lack of a realistic integration method.

A thorough understanding of individual omic platform properties and their influence on data integration efforts represents an important, but usually ignored, aspect of multi-omic experiment planning. Several tools are available to assess omic data quality, even cross-platform, such as FastQC for raw sequencing (seq) reads^14^, Qualimap^15,16^ and SAMstat^17^ for mapping output, and MultiQC^18^ that combines many different tools in a single report. However, these tools do not apply to non-sequencing omics (i.e., metabolomics) and are not conceived to compare platform performance nor support multi-omic experimental design choices. Figures of Merit (FoM) are performance metrics typically used in analytical chemistry to describe devices and methods. FoM include accuracy, reproducibility, sensitivity, and dynamic range; descriptors also applied to omic technologies. However, the definition of each FoM acquires a slightly different specification depending on the omic technology considered, and each omic platform possesses different critical FoM. For example, RNA-seq usually provides unbiased, comprehensive coverage of the targeted space (i.e., RNA molecules), while this is not the case for shotgun proteomics, which is strongly biased toward abundant proteins. Importantly, we currently lack both a systematic description of FoM discrepancies across omic assays and a definition of a common performance language to support discussions on the multi-omic experimental design.

FoM are relevant to statistical analyses that aim to detect differential features, due to their impact on the number of replicates required to achieve a given statistical power. The statistical power of an analysis method, which is the ability of the method to detect true changes between experimental groups, is determined by the within-group variability, the size of the effect to be detected, the significance level to be achieved, and the number of replicates (or observations) per experimental group, also known as sample size. All these parameters are highly related to FoM. Estimating power in omic experiments is challenging because many features are assessed simultaneously^19^. These features may have different within-condition variability and the significance level must be adapted to account for the multiple testing scenario. Deciding on the effect size to detect may also prove difficult, especially when the natural dynamic range of the data has changed due to normalization procedures. Moreover, different omic platforms present distinct noise levels and dynamic ranges, and hence analysis methods might not be equally applicable to all of them. As a consequence, independently computing the statistical power for each omic might not represent the best approach for a multi-omic experiment, if the different measurements are to be analyzed in an integrative fashion and a joint power study for all platforms seems more appropriate. Although several methods have been proposed to optimize sample size and evaluate statistical power in single-omic experiments^19,20^, no such tool exists in the context of multi-omics data. Similarly, multi-omic datasets are increasingly collected to develop sample class predictors applying machine learning (ML) methods. In this case, the classification error rate (ER), rather than the significance value, is used to assess performance. In the field of ML, the estimation of the number of samples required to achieve an established prediction error is still an open question^21,22^ and there are not yet methods that answer this question for multi-omics applications.

In summary, the multi-omics field currently lacks a comparative description of performance metrics across omic technologies and methods to estimate the number of samples required for their multiple applications. In this work, we propose a formal definition of FoM applicable across several omics and provide a common language to describe the performance of high-throughput methods frequently combined in multi-omic studies. We leverage this harmonized quality control vocabulary to develop MultiPower, an approach for power calculations in multi-omic experiments applicable to across omics platforms and types of data. Additionally, we present MultiML, an R method to obtain the optimal sample size required by ML approaches to achieve a target classification ER. The FoM definitions, together with the MultiPower and MultiML calculations proposed here constitute a framework for quality control and precision in the design of multi-omic experiments.

## Results

### Comparative descriptions of omic measurement quality

**Fig. 1:**
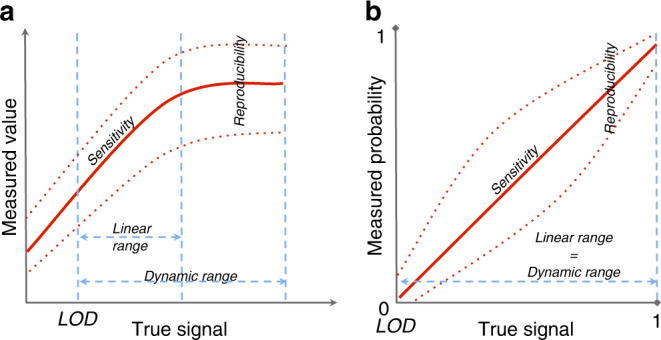
Analytical FoM related to the calibration lines. **a** Calibration line for omics measuring the levels of the target features (MS platforms and gene-based sequencing platforms). **b** Calibration line for omics not measuring concentrations but finding genomic regions, where a biological event occurs (region-based sequencing platforms).

**Fig. 2:**
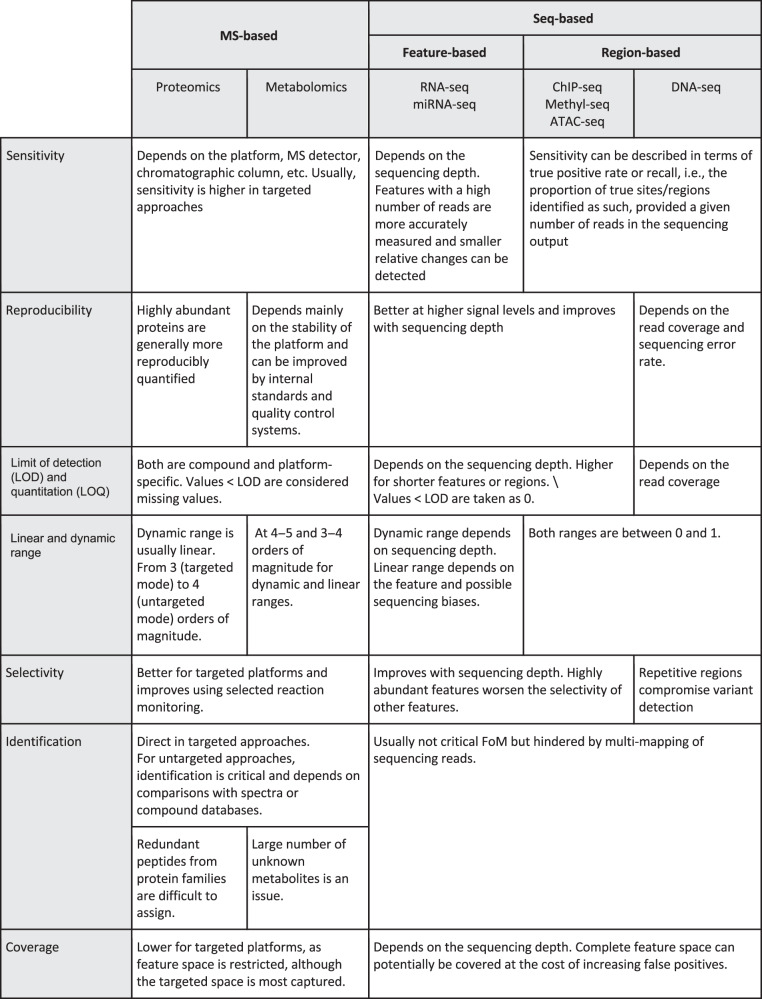
FoM across omic platforms. The table summarizes critical aspects of each FoM and omic.

We selected seven commonly used FoM that cover different quality aspects of molecular high-throughput platforms (see “Methods” section, Fig. 1, Supplementary Table 1). Figure 2 summarizes the comparative analysis for these FoM across seven different types of omic platforms, classified as mass spectrometry (MS) based (proteomics and metabolomics) or sequencing based. In this study, we consider short-read seq-based methods that measure genome variation and dynamic aspects of the genomes, and divided them into feature-based (RNA-seq and miRNA-seq) and region-based methods (DNA-seq, ChIP-seq, Methyl-seq, and ATAC-seq).

Sensitivity is defined as the slope of the calibration line that compares the measured value of an analyte with the true level of that analyte (Fig. 1). For a given platform and feature, sensitivity is the ability of the platform to distinguish small differences in the levels of that feature. Features with low sensitivity suffer from less accurate quantification and are more difficult to be significant by differential analysis methods. In metabolomics platforms, sensitivity primarily depends on instrumental choices, such as the chromatographic column type, the mass detector employed, and the application of compound derivatization^23^. Targeted proteomic approaches sample many data points per protein, leading to higher accuracy when compared to untargeted methods^24^. In nuclear magnetic resonance (NMR), no separation takes place and a low number of nuclei change energy status, leading the detection of only abundant metabolites and lower sensitivity than liquid chromatography (LC)–MS or gas chromatography (GC)–MS. NMR is capable of detecting ~50–75 compounds in human biofluid, with a lower sensitivity limit of 5 µmolar (ref. ^25^), while MS platforms are able to measure hundreds to thousands of metabolites in a single sample. Sensitivity in sequencing platforms depends on the number of reads associated with the feature, a parameter influenced by sequencing depth. Features with an elevated number of reads are more accurately measured, and hence smaller relative changes can be detected. For region-based omics, where the goal is to identify genomic regions where a certain event occurs, the above definition is difficult to apply, and sensitivity is described in terms of true positive rate or recall, i.e., the proportion of true sites or regions identified as such, given the number of reads in the seq output.

Reproducibility measures how well a repeated experiment provides the same level for a specific feature or, when referring to technical replicates, the magnitude of dispersion of measured values for a given true signal. Traditionally, the relative standard deviation (RSD) is used as a measure of reproducibility, with RSD normally differing over the concentration range in high-throughput platforms^26,27^. Generally, reproducibility in sequencing platforms improves at high signal levels, such as highly expressed genes or frequent chromatin-related events. RSD is roughly constant in relation to signal in MS platforms, although in LC–MS the lifetime of the chromatographic column strongly influences reproducibility, leading to small datasets being more reproducible than experiments with many samples. This constraint imposes the utilization of internal standards, quality control samples, and retention time alignment algorithms in these technologies^28^. Moreover, untargeted LC–MS proteomics quantifies protein levels with multiple and randomly detected peptides, each with a different ER, a factor that compromises the reproducibility of this technique. On the contrary, NMR is a highly linear and reproducible technique^29^, and reproducibility issues are associated with slight differences in sample preparation procedures among laboratories. Sequencing methods normally achieve high reproducibility for technical replicates^5^ and are further improved as sequencing depth increases. Reproducibility at the library preparation level depends on how reproducible the involved biochemical reactions are. Critical factors affecting reproducibility include RNA stability and purification protocols in RNA-seq^30^, antibody affinity in ChIP-seq^6^, quenching efficiency in metabolomics^31^, and proteolytic digestion and on-line separation of peptides in proteomics^32,33^. Methyl-seq experiments based on the robust *MspI* enzymatic digestion and bisulfite conversion usually are more reproducible data than enrichment-based methods, such as methylated DNA immunoprecipitation (MeDIP)^34^. Finally, reproducibility for DNA variant calling is associated with the balance between read coverage at each genome position and the technology sequencing errors.

The limit of detection (LOD) of a given platform is the lowest detectable true signal level for a specific feature, while the limit of quantitation (LOQ) represents the minimum measurement value considered reliable by predefined standards of accuracy^35^. Both limits affect the final number of detected and quantified features, which in turn impacts the number of tested features and the significance level when correcting for multiple testing. For MS-based methods, LOD and LOQ depend on the platform, can be very different for each compound, and normally require changes of instrument or sample preparation protocol for different chemicals. Additionally, sample complexity strongly affects LOD, as this reduces the chance of detecting low-abundance peptides, while pre-fractionation can reduce this effect at the cost of longer MS analysis time. NMR has usually higher LOD than MS-based methods. Conversely, LOD depends fundamentally on sequencing depth in seq-based technologies, where more features are easily detected by simply increasing the number of reads. However, there also exist differences in LOD across features in sequencing assays. Shorter transcripts and regions usually have higher LODs and are more affected by sequencing depth choices. For DNA-seq, the ability to detect a genomic variant is strongly dependent on the read coverage. MS-based and seq-based methods also differ in the way features under LOD are typically treated. MS methods either apply imputation to estimate values below the LOD (considered missing values)^12^, or exclude features when repeatedly falling under the LOD. In sequencing methods, LOD is assumed to be zero and data do not contain missing values, although, also in this case, features with few counts in many samples risk exclusion from downstream analyses.

The dynamic range of an omic feature indicates the interval of true signal levels that can be measured by the platform, while the linear range represents the interval of true signal levels with a linear relationship between the measured signal value and the true signal value (Fig. 1). These FoM influence the reliability of the quantification value and, consequently, the differential analysis, as detection of the true effect size depends on the width of these ranges.

In proteomics, molecule fragmentation by data-independent acquisition approaches increases the dynamic range by at least two orders of magnitude. A typical proteomic sample covers protein abundance over 3–4 to four orders of magnitude, a value that increases for targeted approaches^36,37^. In metabolomics, linear ranges usually span 3–4 orders of magnitude, while dynamic ranges increase to 4–5 orders and can be extended using the isotopic peak of the analytes. A combination of analytical methods can increase the dynamic range, as different instruments may better capture either high or low concentration metabolites. NMR has a high dynamic range and can measure highly abundant metabolites with precision, although it is constrained by a high detection limit. For feature-based sequencing platforms, the dynamic range strongly depends on sequencing depth, and values can range from zero counts to up to hundreds of thousands, or even millions (in RNA-seq, for some mitochondrial RNA transcripts). However, due to technical biases, linear range boundaries are difficult to establish and may require the use of calibration RNAs (spike-ins). Moreover, linear ranges may be feature dependent and affected by sequence GC content, which then requires specific normalization. For region-based sequencing platforms, linear and dynamic ranges coincide, and vary from zero to one.

A platform displays good selectivity if the analysis of a feature is not disturbed by the presence of other features. Selectivity influences the number and quantification of features and, consequently, statistical power. For MS platforms, targeted rather than untargeted methods obtain the best selectivity. In metabolomics, selected reaction monitoring, a procedure that couples two sequential MS reactions to obtain unique compound fragments and control quantitation bias using isobaric compounds^38^, is used to improve selectivity, while in proteomics selectivity strongly depends on sample complexity, which determines whether a given feature is detected or not. Similarly, in sequencing experiments, selectivity relates to competition of fragments to undergo sequencing. Highly abundant features or regions (e.g., a highly expressed transcript or a highly accessible chromatin region) may outcompete low-abundant elements. Generally, this problem is difficult to address by means other than increasing the sequencing depth. In the case of transcriptomics, the application of normalized libraries can partially alleviate selectivity problems; however, at the cost of compromising expression level estimations. Particularly for DNA-seq, variant detection is compromised at repetitive regions and can be alleviated with strategies that increase read length.

Identification refers to the relative difficulty faced when determining the identity of the measured feature, a critical issue in proteomics and metabolomics. In principle, identification does not affect power calculations, but influences downstream integration and interpretation. Metabolite identification in untargeted MS-based methods requires comparisons with databases that collect spectra from known compounds^39–41^. Compound fragments with similar masses and chemical properties often compromise identification, while database incompleteness also contributes to identification failures. A typical identification issue in lipidomics is that many compounds are reported with the same identification label (i.e., sphingomyelins), but slightly different carbon composition and unclear biological significance. Conversely, NMR is highly specific. Each metabolite has a unique pattern in the NMR spectrum, which is also often used for identification of unknown compounds. For untargeted proteomics data, either spectral or sequence databases are used to determine the sequence of the measured peptide. Current identification rates lie at ~40–65% of all acquired spectra with a false discovery rate (FDR) of 1–5%. In targeted approaches, identification is greatly improved by the utilization of isotopically labeled standards. Proteomics suffers from the additional complexity of combining peptides to identify proteins, which is not straightforward. In fact, a recent study highlighted identification as one of the major problems in MS-based proteomics due to differences in search engines and databases^42^ and to high false-positive rates^43^. A common identification problem in seq-based methods is the difficulty to allocate reads with sequencing errors or multiple mapping positions, which is addressed either by discarding multi-mapped reads or by estimating correct assignments using advanced statistical tools^44,45^. In ChIP-seq, an identification-related issue is the specificity of the antibody targeting the protein or epigenetic modification. Low antibody specificity may result in reads mapping to nonspecific DNA sequences, leaving true binding regions unidentified. The ENCODE consortium has developed working standards to validate antibodies for different types of ChIP assays^6^.

The coverage of a platform is defined here as the proportion of detected features in the space defined by the type of biomolecule (aka feature space). Targeted MS platforms measure a small subset of compounds with high accuracy; hence the coverage is restricted and lower than for untargeted approaches. As sample complexity is frequently much larger than the sampling capacity of current instruments, even in untargeted methods, identification is limited to compounds with the highest abundances. Repeating sample measurement excluding the features identified in the first run is an efficient strategy to improve coverage, although this requires increased instrument runtime and sample amounts. Coverage in seq-based methods strongly depends on sequencing depth and can potentially reach the complete feature space associated with each library preparation protocol. In RNA-seq, oligodT-based methods recover polyA RNAs, whereas total RNA requires ribo-depletion, capture of antisense transcripts imposes strand-specific protocols, and microRNAs require specific small RNA protocols. In Methyl-seq, coverage also relates to the applied protocol. Whole-genome bisulfite sequencing has greater genome-wide coverage of CpGs when compared to Reduced Representation by Bisulfite Sequencing (RRBS), while RRBS and MeDIP provide greater coverage at CpG islands. In general, region-based sequencing approaches can cover the whole reference genome, with coverage depending on the efficiency of the protocol employed to enrich the targeted regions.

### From FoM to experimental design

**Fig. 3:**
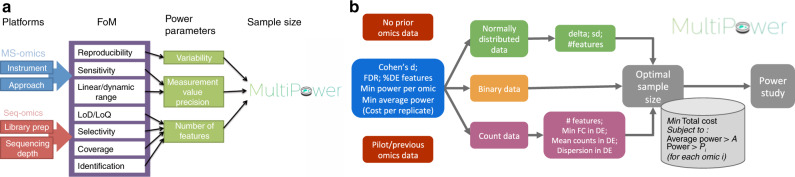
The MultiPower approach. **a** Relationship between omic platforms (MS in blue and sequencing in red), FoM (purple), power parameters (green), and MultiPower. MS- and seq-based omics have different key properties that determine their FoM, which in turn affect differently to power parameters used as input by MultiPower to compute sample size in multi-omics experiments. **b** Overview of the MultiPower method. MultiPower works either with no prior data and with pilot data (red boxes). The blue box contains the parameters to be set by users. Green, orange, and purple boxes represent the data types accepted by MultiPower and the rest of power parameters estimated by MultiPower from prior data when available or given by the user. MultiPower solves an optimization problem to estimate the optimal sample size and to provide a power study of the experiment (gray boxes).

The FoM analysis across omics revealed that each omic data type possesses different critical performance metrics. In MS, FoM mainly depend on the choice of instrument and approach—targeted vs. untargeted—, while FoM in sequencing methods rely on sequencing depth, library preparation, and eventual bioinformatics post-processing. Although FoM are not a property of data but of the analytical platform, they directly impact data characteristics relevant to experimental design. Overall, FoM strongly relate to the variability in the measurements, which changes in a feature-dependent manner. FoM also determine the final number of measured features and the magnitude of detectable change. Hence, reproducibility influences measurement variability; sensitivity, linear, and dynamic range determine the magnitude and precision of the measurements, and are associated to effect size; the number of features measured by the omic platform is given by the LOD, selectivity, identification, and coverage. These three parameters, variability, effect size, and number of variables, are the key components of power calculations in high-throughput data used by MultiPower to estimate sample size in multi-omic experiments. Figure 3a illustrates the relationship between platform properties, FoM, power parameters, and MultiPower, while Fig. 3b presents an overview of the MultiPower algorithm (see “Methods” section for details).

### MultiPower estimates sample size for a variety of experimental designs

**Fig. 4:**
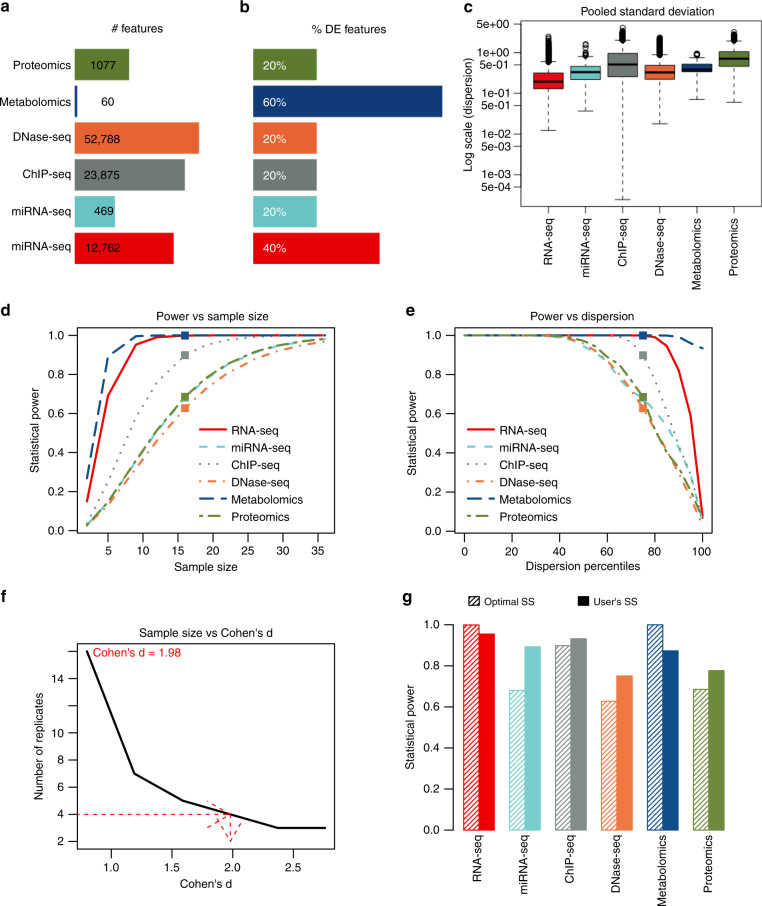
MultiPower application to STATegra data. **a**–**c** Pilot data analysis. Each color represents a different omic data type. **a** Number of features per omic. **b** Expected percentage of differentially expressed (DE) features for each omic. **c** Pooled standard deviation (PSD) per gene and per omic for the estimated DE features (pseudo-DE features, see “Methods” section for details). Boxplots represent the median and interquartile range (IQR). Whiskers depict the minimum and maximum of data without outliers, which are the values outside the interval (Q1 − 1.5 × IQR, Q3 + 1.5 × IQR). **d**–**g** MultiPower results with parameters same sample size in all the omics, minimum power per omic = 0.6, minimum average power = 0.8, FDR = 0.05, initial Cohen’s *d* = 0.8. **d** Statistical power curves for each omic for sample sizes between 2 and 35. Squared dots indicate power at the optimal sample size (*n* = 16). **e** Statistical power curves for each omic when considering different percentiles of PSD. Squared dots indicate power for the dispersion used in power calculations for each omic (75th percentile). **f** Curve relating the initial Cohen’s *d* to the optimal sample size needed to detect each magnitude of change. For the specified sample size (*n* = 4), the red arrows and text highlight the magnitude of change to be detected (Cohen’s *d* = 1.98 in this case). **g** Statistical power per omic using the optimal sample size (*n* = 16) with Cohen’s *d* = 0.8, and the maximum sample size allowed by the user (*n* = 4) with Cohen’s *d* = 1.98.

We applied MultiPower to the STATegra data^46^ (Supplementary Note 1) to illustrate power assessment of an existing multi-omic dataset. Figure 4 compares the number of features (*m*), expected percentage of features with a significant signal change (*p*_1_), and variability measured as pooled standard deviation (PSD). Note that the number of features measured by each omic platform varies by several orders of magnitude, from 60 metabolites to 52,788 DNase-seq regions (Fig. 4a). This, together with the expected percentage of differentially abundant features (Fig. 4b), affects statistical power when multiple testing correction is applied. Given that PSD is different for each feature (Fig. 4c), users can set the percentile of PSD for power estimations (see “Methods” section). In this example, PSD equals the third quartile, which is a conservative choice. We used MultiPower to calculate the optimal number of replicates for each omic imposing a minimum power of 0.6 per technology, an average power of at least 0.8, an FDR of 0.05, an initial Cohen’s *d* of 0.8, and same sample size across platforms. MultiPower estimated the optimal number of replicates to be 16 (Fig. 4d, Table 1), which is the number of replicates required by DNase-seq to reach the indicated minimum power. Power estimates were lowest for DNase-seq, followed by proteomics and miRNA-seq, while features with variability below the *P*_60_ percentile, DNase-seq, proteomics, and miRNA-seq displayed power values above 0.8 (Fig. 4e). The power plots also indicated that metabolomics and RNA-seq data had the highest power, implying that  the detection of differentially expressed (DE) features for these omics is expected to be easier. As costs for generating 16 replicates per omic might be prohibitive, alternatives can be envisioned such as allowing a different number of replicates per omic—at the expense of sacrificing power in some technologies—, accepting a higher FDR, or detecting larger effect sizes. For instance, with four replicates per condition and omic, significant changes were detected at a Cohen’s *d* of 1.98 (Fig. 4f). Figure 4g depicts a per omic summary of the effective power at the optimal sample size (*n* = 16) with a Cohen’s *d* of 0.8, and at a sample size (*n* = 4) with a larger Cohen’s *d*. Results showed that a reduction in the number of replicates can counteract the loss of power if accepting an increase in the magnitude of change to be detected. Finally, MultiPower estimates were further validated by calculating power and Cohen’s *d* with the published replicate numbers in STATegra (*n* = 3), and verifying the agreement in magnitude and direction of change between RNA-seq and RT-PCR for six B-cell differentiation marker genes^46^ (Supplementary Fig. 1).

**Table 1:** MultiPower parameters and results from the STATegra pilot data.

Omic	numFeat^b^	DEperc^b^	Delta^a^	Dispersion^a^	minSampleSize	optSampleSize	Power
RNA-seq	12,762	0.4	0.61	0.32	5	16	0.999
miRNA-seq	469	0.2	0.50	0.46	14	16	0.680
ChIP-seq	23,875	0.2	1.35	0.96	10	16	0.898
DNase-seq	52,788	0.2	0.51	0.49	16	16	0.627
Metabolomics	60	0.6	1.20	0.52	4	16	1.000
Proteomics	1077	0.2	1.16	1.05	14	16	0.685

MultiPower results were obtained for the same sample size in all technologies, a minimum power per omic of 0.6, a minimum average power of 0.8, and a Cohen’s *d* of 0.8.

*numFeat* number of omic features, *DEperc* expected proportion of DE features, *delta* difference of means to be detected, *dispersion* pooled standard deviation, *minSampleSize* sample size to achieve the minimum power per omic, *optSampleSize* optimal sample size for the experiment, *power* power reached with the optimal sample size.

^a^Parameter estimated by MultiPower.

^b^Parameter provided by the user.

Experimental designs with different sample sizes per omic may limit statistical analysis options, but might be unavoidable or preferred in certain studies. We assessed this possibility with the STATegra dataset assuming the same cost for each technology and keeping the rest of the parameters identical to the previous example. MultiPower analysis revealed that miRNA-seq and DNase-seq required the highest sample size (*n* = 17), while only six and nine replicates per group were required by metabolomics and RNA-seq, respectively (Supplementary Table 2, Supplementary Fig. 2). Power plots revealed that decreasing the sample size for miRNA-seq, DNase-seq, or proteomics results in a strong reduction in power. Again, an alternative to reducing power is to increase the magnitude of change to detect that was initially set to Cohen’s *d* = 0.8 (Supplementary Fig. 2c). For example, for a sample size not higher than *n* = 5, the graph indicates a Cohen’s *d* of 1.59 for all omics. Additionally, MultiPower can also handle different costs per omic platform and use this information to propose larger sample sizes for inexpensive technologies (Supplementary Tables 3 and 4, Supplementary Fig. 3).

Human multi-omic cohort studies such as The Cancer Genome Atlas database (TCGA) usually collect data from a large number of subjects, where biological variability is naturally higher than in controlled experiments. In such studies, not all subjects may have measurements at all omic platforms and when integrating data decisions should be made to either select individuals profiled by all omic assays—to keep a complete multi-omic design—or to allow a different number of individuals per platform. We illustrate the utility of our method in cohort studies by using MultiPower to estimate power for the integrative analysis of four omic platforms available for the TCGA Glioblastoma dataset^47^ (Supplementary Note 1). MultiPower indicated that *n* = 24 (Supplementary Table 5, Supplementary Fig. 4) is the optimal sample size for complete designs. In this case, Methyl-seq set the required sample size due to the high number of features, low expected percentage of DE features and high variability of this dataset. As the optimal sample size is similar to the number of samples available in the less prevalent omics modality (only 22 samples are available for proneural tumor in methylation data), the joint analysis of current data is not expected to suffer from a major lack of power (Supplementary Fig. 5). However, smaller sample sizes would dramatically impact the number of detected DE features (Supplementary Fig. 5), further validating the results of the MultiPower method. Given that power is affected by the number of omic features (Fig. 3a), an alternative to adjusting power here is the exclusion of methylation features with low between-group variance, as this reduces the magnitude of the multiple testing correction effect on the loss of power. MultiPower helps to assess these options. For example, keeping only methylation sites with an absolute log2 fold change >0.05 for the power analysis resulted in a reduction of the optimal sample size from 24 to 22 (Supplementary Table 6).

### MultiML predicts sample size for multi-omic based predictors

**Fig. 5:**
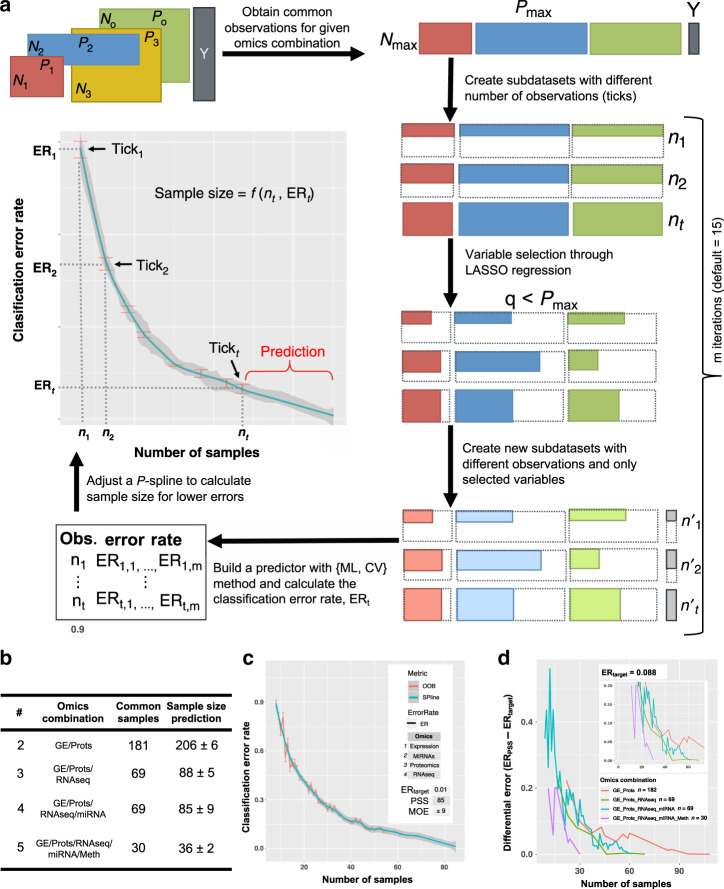
The MultiML algorithm. **a** Given a multi-omic dataset with *O* different omic types and *N*_*O*_ samples per omic and a classification vector Y, MultiML obtains the maximum number of common observations *N*_max_ (*N*_max_ ≤ *N*_*O*_) for the combination of omic types specified by the user. MultiML then creates subdatasets (ticks) having different number of observations (*n*_*t*_) from the available *N*_max_ and obtains the variables that best explain each tick through LASSO regression. Taking provided machine learning algorithm ML and cross-validation method CV, the selected variables at each tick are used to predict the classification error rate (ER) on a different random subdatasets of size *n*_*t*_, resulting in ER_*t*._ This variable selection-ER prediction process is repeated through *m* iterations to obtain an average ER and confidence interval for each tick. On these, *n*_1_ to *n*_*t*_ and ER_1_ to ER_*t*_ values, a first-order-smoothed penalized P-spline regression is adjusted to estimate the learning curve that will allow the prediction of classification ERs for sample sizes larger than *N*_max_. **b**–**d** MultiML results on the TCGA Glioblastoma data. **b** Predicted sample size for different omics combinations using a target ER of 0.01. Margin of error is given as number of samples. GE expression microarrays, Prots proteomics, miRNA-seq microRNA microarrays, Meth methylation microarrays. **c** Example of MultiML graphical output with the predictive P-spline for a combination of four omics types. OOB out-of-bag classification ER, ER_target_ target error rate, PSS predicted sample size, MOE margin of error. **d** MultiML evaluation. An increasing number of observations and omic types were run as input data in MultiML having as ER_target_, the ER achieved with complete data for four omics (69 observations). For the PSS, their ER are recovered from the actual data and their deviation from ER_target_ is calculated as ΔError. The ΔError is plotted against the size of the input data. Accuracy in ER predictions increases with the size of input data and the number of omics.

Multi-omics datasets may be used in cohort studies to classify biological samples into, for instance, disease subtypes or to predict drug response. In these cases, the analysis goal is not to detect a size effect but to achieve a specified prediction accuracy. MultiML computes the optimal sample size for this type of problem. Briefly, MultiML uses a pilot multi-omics dataset to estimate the relationship between sample size and prediction error, which is then used to infer the sample size required to reach a target classification ER (Fig. 5a, “Methods” section). MultiML is a flexible framework that (i) predicts sample sizes using different combinations of omics platforms to obtain the best predictive set, as omics technologies may have similar or complementary information content for a given classification scenario; (ii) accepts user-provided machine learning algorithms beyond the implemented partial least squares discriminant analysis (PLS-DA) and random forest (RF) methods; and (iii) offers job parallelization options to speed up calculations when high-performance computing resources are available.

We illustrate MultiML performance using TCGA Glioblastoma data^47^. Omic features were used to predict tumor subtypes with RF, and the target classification error rate (ER_target_) was set to 0.01. Sample sizes were calculated for combinations of two to five omics platforms. We found that the number of samples required to obtain ER_target_ decreased as the number of omics data platforms increased, suggesting that complementary information was captured by the multi-omics approach resulting in a more efficient predictor (Fig. 5b). Figure 5c shows the classification ER curve fitted by MultiML for a predictor with four omics. A quadratic pattern is observed, where ERs rapidly decrease as the number of samples increases to reach a stable classification performance. This graph can be used to calculate the number of samples required at different ER levels.

To validate the estimations given by MultiML, we mimicked a prediction scenario where we took fractions of the Glioblastoma data and used MultiML to estimate the sample size for ER_target_ equal to the ER of the complete dataset (MinER) or greater. Then, for the predicted sample sizes, we obtained their actual ER from the data and compared them to ER_target_ to evaluate the accuracy of the MultiML estimate, and if the magnitude of the input dataset affected this accuracy. As expected, MultiML accuracy increased with the size of the input data and the number of included omics types (Fig. 5d). Accurate predictions (deviations < 5%) were obtained with 50 samples in a three omics combination. Results were similar for other values of ER_target_ (Supplementary Fig. 6). We concluded that the sample size could be accurately predicted when input data represents ~40–60% of the required sample size.

## Discussion

As multi-omic studies become more common, guidelines have been proposed for dealing with experimental issues (i.e., sample management) associated with specific omics data types^48^. However, the field has yet to address aspects that are essential to understand the complexity of multi-omic analysis, such as the definition of performance parameters across omic technologies and the formulation of an experimental design strategy in multi-omic data scenarios. In this study, we addressed both issues by proposing FoM as a language to compare omic platforms, and by providing algorithms to estimate sample sizes in multi-omic experiments aiming at differential features analysis (MultiPower) or at sample classification using ML (MultiML).

FoM have traditionally been used in analytical chemistry to describe the performance of instruments and methods. These terms can also be intuitively applied to sequencing platforms, but we noticed that the meaning and relevance of FoM slightly differ for both types of technologies. Here, we explain FoM definitions across omic platforms and discuss which of these metrics are critical to each data type. Detection limit, selectivity, coverage, and identification are FoM with critical influence on the number of features comprising the omics dataset, which in turn affects the power of the technology to identify features with true signal changes. Power diminishes as the number of features increases due to the application of multiple testing corrections to control false positives. Reproducibility and dynamic range may also be very different across platforms, and these have a direct impact on the within-condition variability and across-condition differences of the study. Moreover, while sequencing depth critically affects many of the described FoM of sequencing platforms, in MS-based methods, the choice of a targeted or untargeted method strongly influences FoM values. The number of features detected by the omic platform together with the different measurement variabilities across features and the magnitude of change to be detected, represent major components of power calculations for omics data. The highly heterogeneous nature of these factors across omics platforms calls for specific methods for power calculations in multi-omic experiments, which is addressed by MultiPower.

MultiPower solves the optimization problem of obtaining the sample size that minimizes the cost of the multi-omic experiment, while ensuring both a required power per omic and a global power. As any power computation approach, MultiPower indicates the sample size required to detect a targeted effect size given a significance threshold. MultiPower graphically represents the relationship between sample size, dispersion, and statistical power of each omic, and facilitates exploration of alternative experimental design choices. Estimates for MultiPower parameters are optimally calculated from pilot data, although they can also be manually provided. The tool accepts normally distributed, count and binary data to facilitate the integration of omic technologies of different analytical nature. Data should have been properly preprocessed and eventual batch effects, removed. In this study, we showcase MultiPower functionalities using sequencing, microarray, and MS data, although the method could be applied to other technologies, such as NMR. Importantly, the optimal sample size can be computed under two different requirements: an equal sample size for all platforms ensuring a common minimal power, or different sample size per omic to achieve the same power. This is relevant for the choice of downstream statistical analysis. Methods that rely on co-variance analysis typically require uniform sample sizes and MultiPower will provide this while revealing the differences in power across data modalities. Methods that combine data based on effect estimates allow sample size differences and can benefit from the equally powered effect estimation. We illustrate the MultiPower method in three scenarios, where different parameterizations are assessed and include both controlled laboratory experiments and cohort data to highlight the general applicability of the method. By discussing interpretations of power plots and the factors that contribute to sample size results, we provide a means to make informed decisions on experimental design and to control the quality of their integrative analysis.

The MultiPower approach is not directly applicable to ML methods used for sample classification, as in this case the basic parameters of the power calculation—significance threshold and effect size—are not applicable. Still, sample size estimation is relevant as multi-omic approaches are frequently used to build classifiers of biological samples. This is not a trivial problem because, aside from FoM, feature relationships within the multivariate space are instrumental in ML algorithms. The MultiML strategy calculates samples size for multi-omic applications, where the classification ER can be used as a measure of performance. MultiML is itself a learning algorithm that learns the relationship between sample size and classification error, and uses this to estimate the number of observations required to achieve the desired classification performance. Hence, pilot data are a requisite for MultiML. Additionally, MultiML has been designed to be flexible for the ML algorithm and to evaluate multiple combinations of omics types in order to identify optimized multi-omic predictors.

Altogether this work establishes, for the first time, a uniform description of performance parameters across omic technologies, and offers computational tools to calculate power and sample size for the diversity of multi-omics applications. We anticipate MultiPower and MultiML will be useful resources to boost powered multi-omic studies by the genomics community.

## Methods

### Scope of the FoM analysis

In this study, we discuss seven FoM, which we broadly classified into two groups: quantitative or analytical FoM include sensitivity, reproducibility, detection and quantification limit, and linear or dynamic range, and qualitative FoM, which are selectivity, identification, and coverage. To describe how they apply to omic technologies, we distinguish between MS and seq-based platforms.

MS platforms refer to metabolomics and proteomics, which often operate in combination with LC–MS or GC–MS. MS platforms can be used for “untargeted” (measuring a large number of features including novel compounds) and “targeted” assays. While MS is the platform used in most proteomics and metabolomics based studies, NMR (refs. ^29,49^) is also a relevant platform in metabolomics and is incidentally discussed.

For sequencing platforms, we also consider two subgroups: “feature-based” and “region-based”. In feature-based assays (i.e., RNA-seq or miRNA-seq), a genome annotation file defines the target features to be quantified, and hence these are known a priori. For region-based assays (i.e., ChIP-seq or ATAC-seq), the definition of the target feature to be measured (usually genomics regions) is part of the data analysis process. Applications of RNA-seq to annotate genomes could be considered a region-based assay. We consider here omic assays with a dynamic component regarding the genotype, such as RNA-seq, ChIP-seq, ATAC-seq, Methyl-seq, proteomics, and metabolomics, and also genome variation analyses. However, single-cell technologies are not included, as they require a separate discussion.

Analytical FoM quantify the quality of an analytical measurement platform and are defined at the feature level (gene, metabolite, region, etc.). We describe FoM as properties of a calibration line that displays the relationship between the measured value and the true quantity of the feature in the sample (Fig. 1). In our case, the definition of the true signal differs between omic platforms. As MS platforms and feature-based seq-based platforms measure the concentration or level of the feature of the gene, protein, or metabolite of interest, the true signal represents the average level across all cells contained in the sample (Fig. 1a). In contrast, region-based sequencing techniques aim to identify the genomic region, where a given molecular event occurs, such as the binding of a transcription factor. In this case, the true signal represents the fraction of cells in the sample (or probability), where the event actually occurs within the given coordinates (Fig. 1b).

Lastly, while analytical FoM are defined at the feature level, omic platforms by nature measure many features simultaneously and consequently, the FoM may not be uniform for all of them. For example, accuracy might be different for low vs. highly expressed genes or polar vs. apolar metabolites. In this study, we consider FoM globally and discuss how technological or experimental factors affect the FoM of different ranges of features within the same platform.

### Overview of MultiPower method

The MultiPower R method (Fig. 3b) performs a joint power study that minimizes the cost of a multi-omics experiment, while requiring both a minimum power for each omic and an average power for all omics. MultiPower calculations are defined for a two groups contrast and implemented in the R package to support the application of the method to single and multiple pairwise comparisons. The parameters required to compute power can be estimated from multi-omic available data (pilot data or data from previous studies) or, alternatively, users can set them. The method considers multiple testing corrections by adjusting the significance level to achieve the indicated FDR. Additionally, MultiPower accepts normally distributed data, count data or binary data, and optimal sample size (number of replicates or observations per condition) can be computed either requiring the same or allowing different sample sizes for each omic. In the latter, the monetary cost is considered as an additional parameter in the power maximization problem. MultiPower can be used to both design a new multi-omic experiment and to assess if an already generated multi-omic dataset provides enough power for statistical analysis.

MultiPower minimizes the total cost of the multi-omics experiment while ensuring a minimum power per omic (*P*_*i*_) and a minimum average power for the whole experiment (*A*). Equation (1) describes the optimization problem to be solved to estimate the optimal number of biological replicates for each omic (*x*_*i*_):

1 $$\begin{array}{l}{\mathrm{min}}\mathop {\sum}\limits_{i = 1}^I {2c_ix_i} \\ {\mathrm{subject}}\,{\mathrm{to}}\!\!:\\ f\left( {x_i,\alpha , \ldots } \right) \ge P_i \quad\forall i = 1, \ldots ,I\\ \frac{{\mathop {\sum }\nolimits_{i = 1}^I f\left( {x_i,\, \alpha , \ldots } \right)}}{I} \ge A\\ x_i \in {\Bbb Z}^ + \end{array}$$

Where *I* is the number of omics, *c*_*i*_ is the cost of generating a replicate for omic *i*, *α* is the significance level, and *f*() represents the power function.

We calculate statistical power under the assumption that the means of two populations are to be compared in the case of count or normally distributed data. Consequently, power is in these cases a function of the sample size (*x*_*i*_) per condition and for a particular omic *i*, the significance level (*α*), and other parameters that depend on the nature of the omic data type. For normally distributed data, a *t*-test is applied and the power for omic *i* is expressed as $f\left( {x_i,\alpha ,\Delta _i,\sigma _i} \right)$, where Δ_*i*_ is the true difference of means in absolute value to be detected, and *σ*_*i*_ is the PSD considering two experimental groups. Count data obtained from sequencing platforms can be modeled as a negative binomial distribution (NB) and an exact test can be applied to perform differential analysis. In this case, the power of omics *i* is estimated as described in ref. ^50^, where power is expressed as $f\left( {x_i,\alpha ,\phi _i,\mu _i,\omega _i} \right)$, being *ϕ*_*i*_ the dispersion parameter of the NB that relates variance and mean (see Eq. (2)). The effect size is calculated with the fold change (*ω*_*i*_) between both groups as well as the average counts (*μ*_*i*_).

2 $$\sigma ^2 = \mu + \mu ^2\phi$$

For binary data with 0/1 or TRUE/FALSE values indicating, for instance, if a mutation is present or not, or if a transcription factor is bound or not, the goal is comparing the percentages of 1 or TRUE values between two populations. In this case, the parameters needed to estimate power are related to the difference between proportions to be detected (the effect size) and the sample size, but variability is not considered.

As MultiPower considers multiple testing correction to control FDR, the significance level given to the power function is adapted to this correction (α*). We followed the strategy proposed in other studies^51–53^ given by Eq. (3).

3 $$\alpha ^ \ast = \frac{{r_1\alpha }}{{(m - m_1)(1 - \alpha )}},$$

where *m* is the number of features in a particular omic, *m*_*1*_ is the number of expected DE features, *r*_*1*_ is the expected number of true detections, and *α* is the desired FDR.

### Sample size scenarios in MultiPower

In multi-omic studies, an assorted range of experimental designs can be found. All omic assays may be obtained on the same biological samples or individuals, which would result in identical replicates number for all data types. However, this is not always possible due to restrictions in cost or biological material, exclusion of low-quality samples, or distributed omic data generation. In these cases, sample size differs among omic types and yet the data are to be analyzed in an integrative fashion. MultiPower contemplates these two scenarios.

Under the first scenario, adding the constraint of equal sample size for all omics (*x*_*i*_ = *x* for all *I* = 1, …, *I*) to the optimization problem in Eq. (1) results in a straightforward solution. First, the minimum sample size required to meet the constraint on the minimum power per omic (*x*_*i*_) is calculated and the initial estimation of *x* is set to *x* = max_I_{*x*_*i*_}. Next, the second constraint on the average power is evaluated for *x*. If true, the optimal sample size is *x*_opt_ = *x*. Otherwise, *x* is increased until the constraint is met. Note that, under this sample size scenario, the cost per replicate does not influence the optimal solution *x*_opt_.

Under the second scenario, allowing different sample sizes for each omic, the optimization problem in Eq. (1) becomes a nonlinear integer programming problem, as the statistical power is a nonlinear function of *x*_*i*_. The optimization problem can be transformed into a 0–1 linear integer programming problem by defining the auxiliary variables $z_n^i$ for each omic *i* and each possible sample size *n* from 2 to a fixed maximum value *n*^*i*^_max_, where $z_n^i = 1$ when the sample size for omic *i* is *n*. The new linear integer programming problem can be formulated as follows:

4 $$\begin{array}{l}{\mathrm{min}}\mathop {\sum}\limits_{i = 1}^I {2c_i} \left( {\mathop {\sum }\limits_{n = 2}^{n_{\mathrm{max}}^i} nz_n^i} \right)\\ {\mathrm{subject}}\,{\mathrm{to}}\!\!:\\ \mathop {\sum}\limits_{n = 2}^{n_{\mathrm{max}}^i} {f_i} \left( n \right)z_n^i \ge P_i \, \, \forall i = 1, \ldots ,I\\ \frac{{\mathop {\sum }\nolimits_{i = 1}^I \mathop {\sum }\nolimits_{n = 2}^{n_{\mathrm{max}}^i} f_i(n)z_n^i}}{I} \ge A\\ \mathop {\sum}\limits_{n = 2}^{n_{\mathrm{max}}^i} {z_n^i} = 1{\quad}i = 1, \ldots ,I\\ z_n^i\, \in \{ 0,1\}\quad\forall i\,\, \forall n\end{array},$$

where *f*_*i*_(*n*) is the power for sample size *n*, given the parameters of omic *i*.

### MultiPower implementation

The MultiPower R package implements the described method together with several functionalities to support both the selection of input parameters required by the method and the subsequent interpretation of the results. The R package and user’s manual are freely available at https://github.com/ConesaLab/MultiPower.

The MultiPower package requires different parameters to compute the optimal sample size. As the choice of parameters can be challenging, MultiPower can estimate them from pilot or similar existing data. The multi-omic pilot data must have two groups and at least two replicates per group. The algorithm assumes data are already preprocessed, normalized, and free of technical biases. Both normally distributed and count data are accepted. We recommend raw count data to be provided for sequencing technologies as MultiPower deals with the sequencing depth bias. However, when count data contains other sources of technical noise, we recommend previous transformations to meet normality (e.g., with log or voom transformation) and indicating MultiPower that data are normally distributed. We also recommend removing low count features from sequencing data. For data containing missing values, these must be removed or imputed before running MultiPower. MultiPower also accepts binary data (e.g., SNP data, ChIP-seq transcription binding data, etc.). In this case, values must be either 0–1 or TRUE/FALSE.

Each data type requires a different sample size computation. For normally distributed data, MultiPower uses the *power.t.test()* function from *stats* R package (based on a classical *t*-test), while the *sample_size()* and *est_power()* functions from R Bioconductor *RnaSeqSampleSize* package^54^ are used for count data, where a NB test is applied. For binary data, the *power.prop.test()* function from stats R package is used. Moreover, MultiPower transforms parameters provided by the user to fit specific arguments required by these functions, in such a way that magnitudes are maintained roughly comparable for all data types.

The power function for normally distributed data depends on the effect size, i.e., the true difference of means to be detected (delta parameter, Δ). The delta parameter depends on the dynamic range of the omic and may be nonintuitive following data preprocessing. On the contrary, the fold change and mean counts are used in count data to estimate power. Therefore, MultiPower uses instead the Cohen’s *d* (*d*_*0*_*)* value to homogenize input parameters. Cohen’s *d* is defined as Δ/*σ* and does not depend on the scale of the data as occurs for the Δ value. Therefore, the same value can be chosen for all omic platforms. Cohen^55^ and Sawilowsky^56^ proposed the classification presented in Supplementary Table 7 to establish a Cohen’s *d* value. MultiPower computes the Cohen’s *d* value for all omic features and applies this value to estimate the set M_1_ of DE features (those with *d* > *d*_0_), which are called pseudo-DE features. We recommend setting the same Cohen’s *d* initial value (*d*_0_) for all the omics although MultiPower allows different values for each one of them.

The equivalent to Cohen’s *d* when comparing proportions in groups A and B is Cohen’s *h*, which can be defined as *h* = |φ_*A*_ *−* φ_*B*_|, where φ_*i*_ = 2 arcsin √_*pi*_, and _*pi*_ is the proportion of 1 or TRUE values in group *i*. Classification in Supplementary Table 7 is also valid for Cohen’s *h*.

The multiple testing correction is applied to each omic analysis (see Eq. (3)), taking the significance level set by the user as FDR value. The user must also provide the expected percentage of DE features (*p*_1_) for each omic. Given the number of features in a particular omic (*m*), the expected number of DE features can be simply represented as *m*_1_ = *mp*_1_. The RnaSeqSampleSize package^50^ estimates the expected number of true detections *r*_1_ for count data. For normal or binary data, *r*_1_ = *m*_1_ is assumed(as in ssize.fdr R/CRAN package^57^).

The intrinsic characteristics of each omic feature may result in different power values for each one of them. To have a unique power estimation per omic, an average power parameter from the distribution of such parameters across pseudo-DE features must be provided. For normally distributed data, the PSD parameter (*σ*) is computed as the percentile *P*_*k*_ of the PSDs for all the pseudo-DE features, where *P*_*k*_ is set by the user (default value *P*_75_). Thus, the Δ estimation should equal the chosen PSD. However, to avoid dependence on a single value, MultiPower evaluates all the pseudo-DE features with PSD between percentiles *P*_*k*−5_ and *P*_*k*+5_ and the *P*_100 − *k*_ of the corresponding Δ values is taken as conservative choice. For count data, MultiPower estimates the parameter *w* that considers the different sequencing depth between samples as *w* = *D*_*B*_*/D*_*A*_, where *D*_*i*_ is the geometric mean of the sequencing depth of the samples in group *i* divided by median of the sequencing depth of all samples (MSD). To compute the rest of parameters, count values are normalized by dividing them by this ratio, that is, the sequencing depth of the corresponding sample divided by MSD. To be consistent with the previous choice for normally distributed data, the variance per condition (*σ*^2^) is also estimated as the percentile *P*_*k*_ of the variances for all pseudo-DE features and conditions. Mean counts (*μ*) are obtained as the percentile *P*_100−*k*_ of mean counts in the reference group (A) corresponding to pseudo-DE features with variance between percentiles *P*_*k*−5_ and *P*_*k*+5_. The dispersion parameter (*ϕ*) is then derived from Eq. (2). Finally, the fold change of pseudo-DE features (*ω*) is estimated as the percentile *P*_100−*k*_ of the fold changes corresponding to pseudo-DE features with a PSD between percentiles *P*_*k*−5_ and *P*_*k*+5_. For binary data, the proportions chosen to estimate power correspond to the *P*_100−*k*_ of pseudo-DE features for Cohen’s *d*, and are stored in the delta output parameter of MultiPower. As variability is not considered for this data type, dispersion power plots are not generated in this case.

Once the power parameters are obtained for each omic and the user sets the minimum power per omic and the average power for the experiment, the optimization problem in Eq. (4) is completely defined and MultiPower makes use of *lpmodeler* and *Rsymphony* R packages to solve it. Note that the application of these packages to solve the problem is only needed when the number of replicates for each omic differs. MultiPower returns a summary table with the provided and/or estimated power parameters, the obtained optimal sample size, and corresponding power per omic.

### Power parameters in the absence of prior data

While parameter estimation from previous data is recommended for MultiPower analysis, this might not always be feasible. In this case, MultiPower requires parameters to be provided by users, which could be challenging. Here, we provide recommendations for critical MultiPower parameters.

The average value for the standard deviation per omic feature and condition partially depends on the reproducibility of omics technology. Overestimating this parameter guarantees that the sample size fits the power needed but may lead to too large sample sizes. According to our experience, a good value for the standard deviation is 1.

Value for the expected proportion of DE features per omic should be set according to results seen in similar studies. A high percentage is expected for cell differentiation processes or diseases like cancer, while small perturbations or other types of diseases may induce fewer changes.

Typical values for the minimum fold change between conditions and the mean of counts for the DE features when using count data are 2 and 30, respectively, but again these values depend on the sequencing depth of the experiment and the magnitude of the expected molecular changes.

### Power study

After obtaining the optimal sample size with MultiPower, some questions may still arise, especially when this sample size exceeds the available budget for the experiment:

- How much reduction of the sample size can we afford without losing too much power?
- If power cannot be decreased but the sample size has to be reduced, how will this reduction influence the effect size to be detected?
- Can we remove any omic platform with negligible changes between conditions, since this platform imposes a too large optimal sample size?

To provide answers to these and similar questions, MultiPower returns several diagnostic plots (see Fig. 4d–g for instance). The power vs. sample size plot shows variations in power as a function of the sample size. The power vs. dispersion plot also displays the power curves but for different dispersion values (PSD in normal data or *ϕ* parameter in count data). In both plots, the power for the estimated optimal sample size or the fixed dispersion value is represented by a square dot.

If the optimal sample size estimated by MultiPower exceeds the available budget, researchers may opt for increasing the effect size (given by the Cohen’s *d*) to be detected and allowing a smaller sample size without modifying the required power. To perform this analysis, the *postMultiPower()* function can be applied, which computes the optimal sample size for different values of Cohen’s *d*, from the initial value set by the user to *d*_max_ = min_*i in I*_{*P*_90_^*i*^(*d*)}, where *P*_90_^*i*^(*d*) is the 90th percentile of the Cohen’s *d* values for all the features in omic *i*. This choice ensures sufficient pseudo-DE features to estimate the rest of the parameters needed to compute power.

### MultiPower for multiple comparisons

Although MultiPower algorithm is essentially defined for a two groups comparison, the MultiPower R package supports experimental designs with multiple groups. Assuming that a pilot dataset is available, the *MultiGroupPower()* function automatically performs all the possible pairwise comparisons (or those comparisons indicated by the user) and returns both a summary of the power and optimal sample size for each comparison, and a numerical and graphical global summary for all the comparisons. In this global summary, the global optimal sample size is computed as the maximum of optimal sizes obtained for the individual comparisons. Therefore, the solution given by MultiPower method does not allow a different sample size for each group. Users must be aware that different optimal sample size could be obtained in this case.

### MultiML method

The MultiML method deals with a multi-omic sample size estimation problem, in which we have prior data consisting of a list of *O* omic data matrices of dimension *N* observations × *P* predictor variables, and a categorical response variable **Y** providing the class each observation belongs to (Fig. 5). The number of observations and variables can differ across omics. MultiML estimates the optimal sample size required to minimize the classification ER. MultiML allows  users to perform analyses for different combinations of the *O* available omics. In each combination, the algorithm selects the common observations across omics, *N*_max_. Next, an increasing number of observations (from two observations per class to the total number of observations *N*_max_) are selected to build a class predictor using the ML algorithm, sampling strategy (SS), and cross-validation (CV) method indicated by the user. The algorithm incorporates a LASSO variable selection step to reduce the number of predictors and computation time. LASSO regression is incorporated at the performance evaluation step to avoid overfitting. This process is repeated 15 times as default, and for each iteration the classification ER of the predictor is computed. These data are used to build a polynomial model that describes the relationship between ER and the number of observations in the multi-omics dataset, which can be then used to estimate the sample size required for a given ER_target_. Supplementary Fig. 7 shows the pseudocode of the MultiML algorithm.

### MultiML implementation

MultiML is implemented as a set of R functions that calculate the classification ERs at increasing number of observations, fit the sample size predictive model and graphically display results. Auxiliary functions are included to prepare multi-omic data and optimize computational requirements. The main function is *ER_calculator()*, which basically takes multi-omic pilot data and returns the estimated sample size. This function requires an ER_target_, which is the maximum classification ER that the user is willing to accept in the study. When not provided by the user, MultiML takes the ER value obtained from the pilot data. Users may select from two CV methods, tenfold and leave-one-out CV, and prediction performance results are averaged across iterations. MultiML can operate with any user-supplied ML algorithm, provided that this is a wrapped R function with input and output formats supported by MultiML. By default, MultiML includes RF and PLS-DA as ML options. For RF the R package *randomForest*^58^ is required. In this method, the classification ER is calculated after constraining all selected omics variables into a wide matrix. For PLS-DA the *mixOmics* R package^59^ is required. In this case, each omics data matrix is reduced to its significant variables and analysis is performed maintaining a three-way *N* × *P* × *O* structure, where *N* are individuals, *P* are the significant variables, and *O* are the different omics in the study. If PLS-DA is selected as ML method, the SSs may or not be balanced, returning either an overall classification ER or a balanced ER calculated on the left-out samples. For RF method, only ER is available as both methods give similar results. MultiML also provides different prediction distances for PLS-DA and RF used to assign a category to samples. For PLS-DA, maximum distance, distance to the centroid, and Mahalanobis distance were implemented. These prediction distances can be defined as a model with *H* components. Given *N*_newInds_ new individuals and their corresponding omic data matrix **X**_**newinds**_, the predicted response variable **Ŷ**_**newinds**_ can be computed as follows:

5 $$\widehat {\mathbf{Y}}_{{\mathbf{newinds}}} = {\mathbf{X}}_{{\mathbf{newinds}}} \times {\mathbf{W}}\left( {{\mathbf{D}}^{\mathbf{T}}{\mathbf{W}}} \right)^{ - {\mathrm{1}}}{\mathbf{B}}$$

where **W** is a *p* (variables) × *H* matrix containing the loading vectors associated with **X**; **D** is a *p* × *H* matrix containing the regression coefficients of **X** on its *H* latent components; and **B** is an *H* × *n* (individuals) matrix containing the regression coefficients of **Y** on the *H* latent components associated to **X**. The predicted scores (**T**_pred_) are computed as:

6 $${\mathbf{T}}_{{\mathbf{pred}}} = {\mathbf{X}}_{{\mathbf{newinds}}} \times {\mathbf{W}}\left( {{\mathbf{D}}^{\mathbf{T}}{\mathbf{W}}} \right)^{ - {\mathbf{1}}}$$

In turn, for RF, out-of-bag (OOB) estimate was included^60^. The RF classifier is trained using bootstrap aggregation, where each new tree is fit from a bootstrap sample of the training observations *z*_*i*_ = (*x*_*i*_*, y*_*i*_). The OOB error is the average error for each *z*_*i*_ calculated using predictions from the trees that do not contain *z*_*i*_ in their respective bootstrap sample. The prediction distances are then applied to assign a category to each new sample. To reduce the number of omics variables and computational time, a generalized linear model via penalized maximum likelihood is applied. The regularization path is computed for the LASSO penalty using the *glmnet* R package^61^. This variable selection step is performed on a random selection of *n* observations and repeated as many times as required (15 by default) to retain the *q* variables that best explain the classification vector **Y**. The algorithm then takes a new set of *n*′_*i*_ observations and, uses only the *q* variables to calculate the classification ER using the ML and CV method chosen by the user (Fig. 5). The sample size prediction curve is estimated with the vector of ERs $\overline {{\mathbf{ER}}_{{\mathbf{ti}}}}$ and the vector of number of samples **τ**_**i**_. The accuracy of MultiML depends on the number of ticks obtained to fit the sample size prediction curve. The algorithm starts with a low (5) number of ticks and iteratively increases them until the addition of new ticks does not improve the accuracy of the sample size prediction curve. The algorithm termination protocol implemented in MultiML, forces a stop when at least 12% of the range of values of three consecutive models (ticks addition steps) are equal. Finally, a first-order-smoothed penalized P-spline calculation is performed to model the relationship between the number of samples and the classification ER (refs. ^62,63^). The degrees of freedom of this model are the number of observation subsets evaluated (ticks) minus 1. The model is used to predict the sample size required to obtain a given classification error.

MultiML is a computationally intensive algorithm. The function *RequiredTimeTest()* allows users to estimate the time required to run the full predictive model at the local installation. For users who can benefit from parallelization options, we have implemented the *slurm_creator()* R function that creates a. sh script to run MultiML calculations in a SLURM cluster. Moreover, MultiMIL can be run incrementally. The function *Previous_CER()* allows the utilization of a previous MultiML result with *N* samples in a new MultiML calculation that expands the size of the prior dataset by *M* samples, thereby significantly accelerating the calculation of the new model.

### MultiML output

MultiML returns all numerical data of the ML models created to fit the sample size prediction model. This includes the evaluated subsets of observations and data types, the classification ER values obtained at each iteration, and the predicted sample size together with the margin of error of the prediction. Additionally, the function *ErrorRatePlot()* prints graphically the relationship between different sample sizes and their corresponding classification ERs. An example is shown in Fig. 5c. The plot also includes the fitted penalized smooth spline model to graphically obtain the sample size for an ER_target_ not achievable with the pilot dataset. The user can also create a comparative plot of all omic combinations to determine the best contributing data modality to an accurate classification by using *Comparative_ERPlot()* function.

### Reporting summary

Further information on research design is available in the Nature Research Reporting Summary linked to this article.

## Supplementary information

Supplementary Information

Reporting Summary

## Data Availability

The STATegra data used in this manuscript are available from: Gene Expression Omnibus with accession numbers GSE75417, GSE38200, GSE75394, GSE75393, and GSE75390; https://identifiers.org/pride.project:PXD003263; and https://identifiers.org/metabolights:MTBLS283. The TCGA glioblastoma data used in this manuscript are available from https://www.cancer.gov/about-nci/organization/ccg/research/structural-genomics/tcga/studied-cancers/glioblastoma.

## Code availability

The MultiPower and MultiML methods are available at GitHub repository https://github.com/ConesaLab/MultiPower.
