# Supplementary material for: Harmonization of quality metrics and power calculation in multi-omic studies
Source: Nat Commun. 2020 Jun 18;11:3092. doi: 10.1038/s41467-020-16937-8 (PMC7303201; doi:10.1038/s41467-020-16937-8)
Supplement: Supplementary file 1 — Supplementary Information [file 41467_2020_16937_MOESM1_ESM.pdf]

Harmonization of quality metrics and power calculation in multi-omic studies

Sonia Tarazona, Leandro Balzano-Nogueira, David Gómez-Cabrero, Andreas Schmidt, Axel Imhof, Thomas Hankemeier, Jesper Tegnér, Johan A. Westerhuis, Ana Conesa

Supplementary Information

Supplementary notes

Supplementary Note 1

STATegra Data. To demonstrate how MultiPower can help users to assess the quality of their multi-omic dataset in terms of power, we used data generated within the STATegra project[1]. In STATegra, mouse B cell differentiation was studied on B3 cell lines. B3 cells are at a cycling stage and differentiation can be triggered by inducing the expression of the transcription factor Ikaros. Omics measurements were taken at six different time points from 0 to 24 hours, with three biological replicates per condition. The measurements were taken from distinct samples. In this example, we considered a comparison between undifferentiated cells and cells following a 24-hours induction of Ikaros expression and the following omics: RNA-seq, miRNA-seq, ChIP-seq, DNase-seq, proteomics, and metabolomics. Therefore, in this case, the parameters used by MultiPower (number of features, expected differentially expressed features and variability) could be determined from the available data.
We considered the STATegra omic data sets to approximately follow a normal distribution after the pre-processing and transformations applied[1] and we based power calculations on a classical t-test. Thus, the variability was computed as the pooled standard deviation. We derived the expected number of DE features from previous analysis carried on by STATegra consortium data[1].

TCGA Glioblastoma Data. The Glioblastoma study[2] in The Cancer Genome Atlas (TCGA) database (http://cancergenome.nih.gov/) contains different omic data types and platforms. To demonstrate the application of MultiPower to cohort data we selected Gene Expression (Affymetrix array), DNA methylation (Illumina array), miRNA (microarray) and proteomics data for two glioblastoma subtypes: proneural and mesenchymal tumors. Supplementary Table 7 shows the number of samples available for each tumor and omic. The measurements were taken from distinct samples. Supplementary Figure 8 displays the number of features per omic, and the expected percentage of differentially expressed features. We estimated this percentage via the limma R package[3] with a 0.05 threshold for the FDR adjusted p-value. To illustrate the MultiML method, also the Glioblastoma dataset was used but additional cancer subtypes and omic methods were included (Supplementary Table 8).

Supplementary tables

Supplementary Table 1. MultiPower use-case 2: different sample size per omic.

Omic	numFeat	DEperc	delta	dispersion	optSampleSize	Power
**RNA-seq**	12,762	0.4	0.61	0.32	9	0.953
**miRNA-seq**	469	0.2	0.50	0.46	17	0.715
**ChIP-seq**	23,875	0.2	1.35	0.96	14	0.842
**DNase-seq**	52,788	0.2	0.51	0.49	17	0.663
**Metabolomics**	60	0.6	1.20	0.52	6	0.950
**Proteomics**	1,077	0.2	1.16	1.05	16	0.685

MultiPower parameters and results when the optimal sample size differs for each omic (minimum power per omic=0.6 and minimum average power=0.8). Costs considered equal for all omics.

Supplementary Table 2. Cost per replicate.

RNA-seq	miRNA-seq	ChIP-seq	DNase-seq	Metabolomics	Proteomics
c	1.3c	1.5c	1.6c	c	c

Cost of generating a replicate for each omic taking RNA-seq as a reference.

Supplementary Table 3. MultiPower use-case 2: different cost and sample size per omic.

Omic	numFeat	DEperc	delta	dispersion	cost	optSampleSize	Power
RNA-seq	12,762	0.4	0.61	0.32	1.0	9	0.953
miRNA-seq	469	0.2	0.50	0.46	1.3	17	0.715
ChIP-seq	23,875	0.2	1.35	0.96	1.5	13	0.804
DNase-seq	52,788	0.2	0.51	0.49	1.6	16	0.627
Metabolomics	60	0.6	1.20	0.52	1.0	6	0.950
Proteomics	1,077	0.2	1.16	1.05	1.0	18	0.7525

MultiPower parameters and results for STATegra data when the optimal sample size differs for each omic (minimum power per omic=0.6, minimum average power=0.8). The cost differs for each omic (see Supplementary Table 2). MultiPower indicates that increasing the sample size up to n=18 for an inexpensive technology such as Proteomics, maintained the targeted average powr, whie allowing smaller samples sizes for more costly comics such as ChIP-seq and DNase-seq (see also Supplementary Figure 3).

Supplementary Table 4 MultiPower use-case 3: a cohort study.

Omic	numFeat	DEperc	delta	dispersion	minSampleSize	optSampleSize	Power
Expression	12,042	0.64	0.79	0.80	12	24	0.912
Methylation	384,349	0.14	0.83	0.94	24	24	0.625
miRNA	534	0.56	0.87	0.79	10	24	0.960
Proteomics	171	0.44	0.68	0.63	11	24	0.949

MultiPower parameters and results for TCGA glioblastoma data when considering an equal optimal sample size for all omic platforms (minimum power per omic=0.6, minimum average power=0.8).

Supplementary Table 5. MultiPower use-case 3: a cohort study (with methylation filter).

Omic	numFeat	DEperc	delta	dispersion	minSampleSize	optSampleSize	Power
expression	12,042	0.64	0.79	0.80	12	22	0.887
methylation	307,329	0.17	0.83	0.94	22	22	0.603
miRNAs	534	0.56	0.87	0.79	10	22	0.943
proteomics	171	0.44	0.68	0.63	11	22	0.929

MultiPower parameters and results for TCGA glioblastoma data when filtering out methylation sites with absolute log2-fold-change lower than 0.05 (20% of the features). (Optimal sample size equal for all the omics, minimum power per omic=0.6, and minimum average power=0.8).

Supplementary Table 6. Classification for Cohen's d or Cohen's h values

Cohen's d / Cohen's h	Effect size
0.01	Very small
0.2	Small
0.5	Medium
0.8	Large
1.2	Very large
2.0	Huge

Supplementary Table 7. TCGA glioblastoma data used in MultiPower method.

Type of tumor	Gene Expression	DNA Methylation	miRNAs	Proteomics
Mesenchymal	153	31	155	45
Proneural	137	22	134	57

Number of samples per type of tumor and omic.

Supplementary Table 8. TCGA glioblastoma data used in MultiML method.

Type of tumor	Gene Expression	DNA Methylation	miRNAs	Proteomics	RNA-seq
Classical	140	25	138	53	38
Mesenchymal	153	25	150	44	47
Neural	85	11	82	29	25
Proneural	137	21	133	56	36

Number of samples per type of tumor and omic.

Supplementary figures

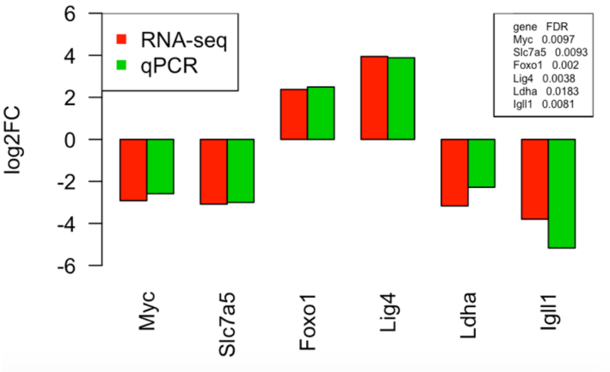

Supplementary Figure 1. MultiPower validation at B-cell markers of STATegra data. Data represent fold change values between Ikaros and Control cells at 24 hours after Ikaros induction, obtained from the STATegra RNA-seq dataset with 3 replicates per condition. Values are compared to qPCR measurements of the same experimental conditions. We used MultiPower to obtain power and Cohen's d for the STATegra dataset nominal samples size (*n*=3), resulting in 0.5 and 2.5. RNA-seq data obtained significant changes for these genes with log2FCs in the range of 2.3 to 3.9, very similar in magnitude and direction to values obtained by qPCR. Although this cannot be considered as a complete validation, these results indicate that biologically validated DE genes were recovered from the data in which they were found as a function of the estimated powers.

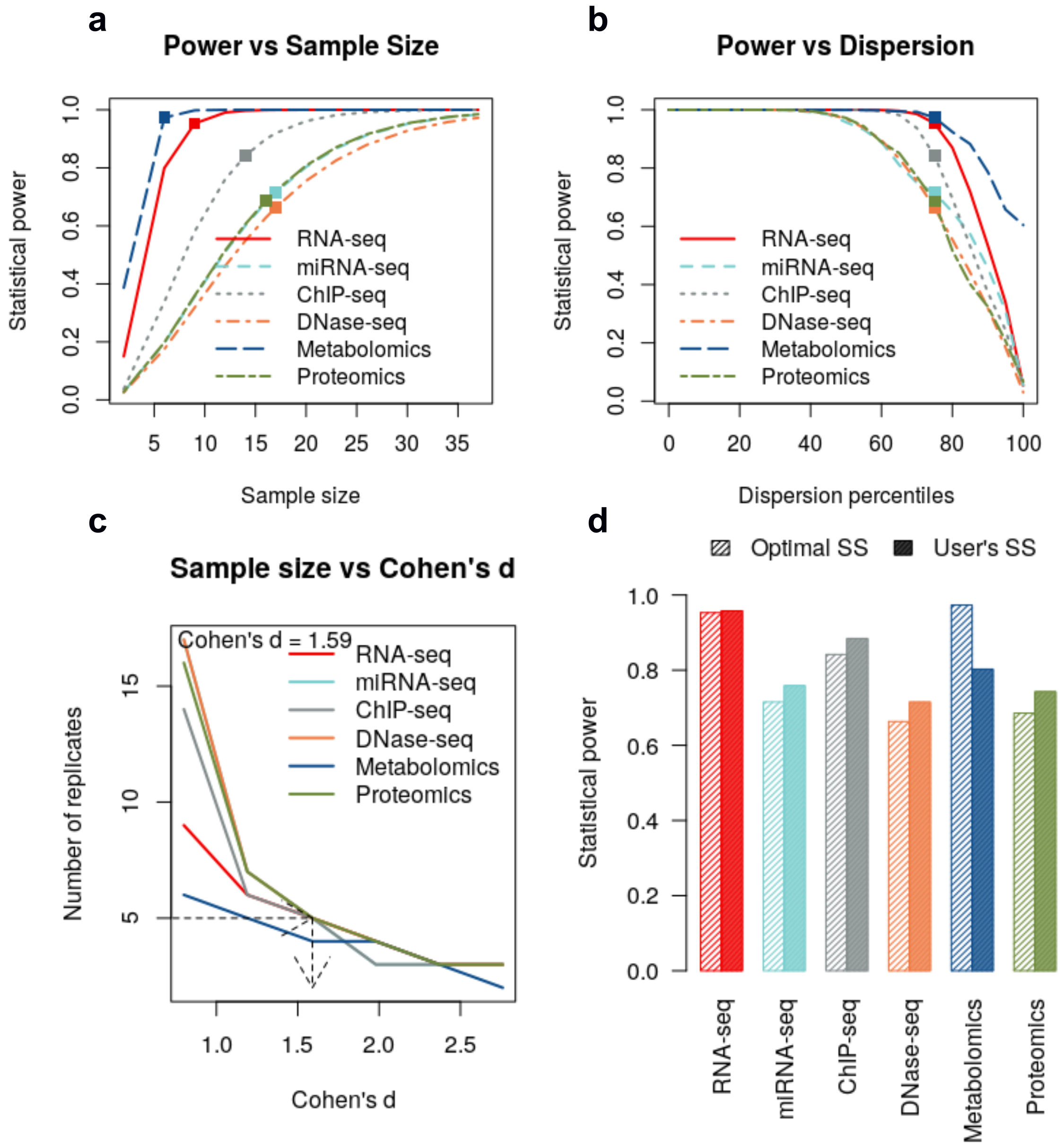

Supplementary Figure 2. MultiPower use-case 2: different sample size per omic**.** Power study for STATegra data allowing for different sample sizes per omic but with the same cost for all omics (minimum power per omic=0.6, minimum average power=0.8). **a** Statistical power curves for each omic when considering different sample sizes. Squared dots indicate power for the optimal sample size for each omic. **b** Statistical power curves for each omic when considering different values of pooled standard deviation. Squared dots indicate power for the estimated dispersion for each omic. **c** Curve relating the initial Cohen's d to the optimal sample size needed to detect each magnitude of change. For the specified sample size (*n*=5), the red arrows and text highlight the magnitude of change to be detected (Cohen's d=1.98 in this case). **d** Statistical power per omic using the optimal sample size obtained for each omic (and initial Cohen's d of 0.8) and the maximum sample allowed by the user (*n*=5), with Cohen's d=1.59.

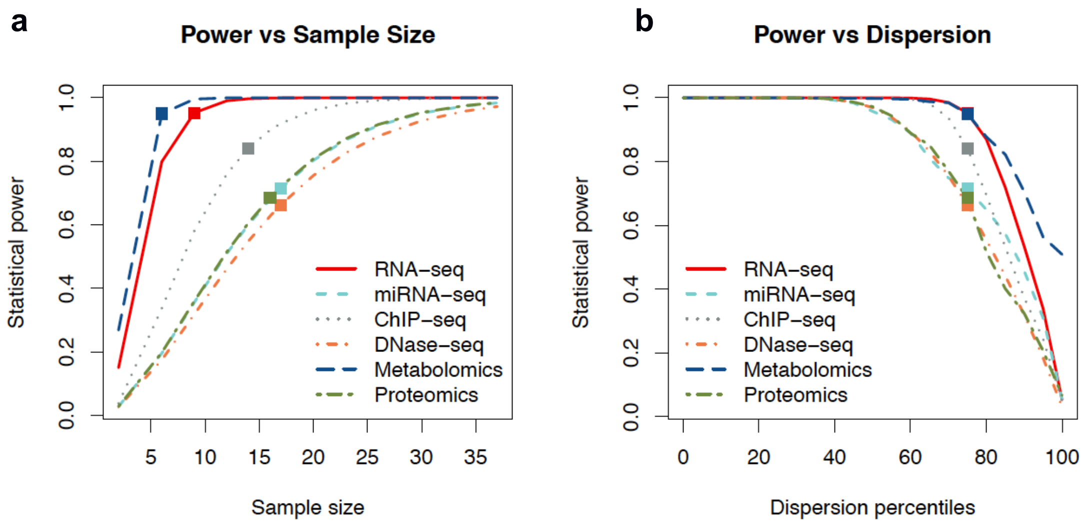

Supplementary Figure 3. MultiPower use-case 2: different sample size per omic**.** Power study for STATegra data when considering differing sample sizes and costs (see Supplementary Table 5) for each omic (minimum power per omic=0.6, minimum average power=0.8). **a** Statistical power curves for each omic when considering different sample sizes. Squared dots indicate power for the optimal sample size for each omic. **b** Statistical power curves for each omic when considering different values of pooled standard deviation. Squared dots indicate power for the estimated dispersion for each omic.

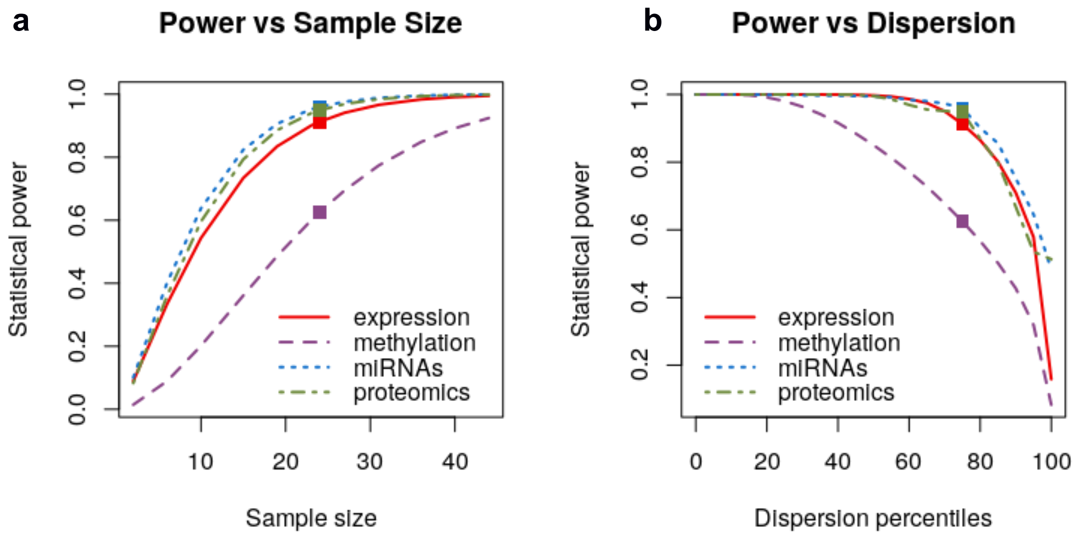

Supplementary Figure 4. MultiPower use-case 3: a cohort study**.** TCGA glioblastoma data. Power study when considering equal sample size for all omics (minimum power per omic=0.6, minimum average power=0.8). **a** Statistical power curves for each omic when considering different sample sizes. Squared dots indicate power for the optimal sample size for each omic. **b** Statistical power curves for each omic when considering different values of pooled standard deviation. Squared dots indicate power for the estimated dispersion for each omic.

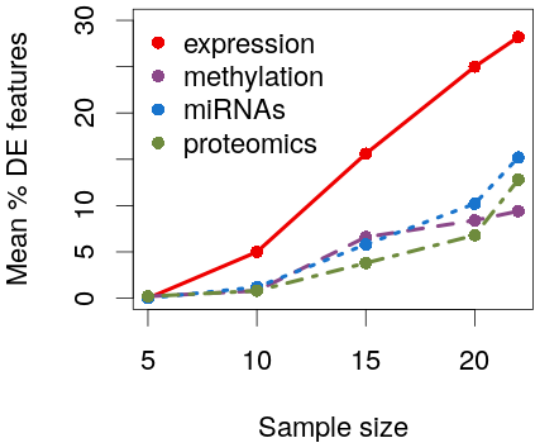

Supplementary Figure 5. MultiPower use-case 3: a cohort study**.** TCGA glioblastoma data. Validation of MultiPower results. Differential expression was computed at different sample sizes including the “quasi” optimal saple size (*n*=5, 10, 15, 20 and 22). For each sample size, data were subsampled 5 times. Percentages of differentially expressed features for the 5 subsamples were averaged. With *n*=5 or *n*=10, no or few differentially expressed features were found for all the omics. Although the true number of differentially expressed features is unknown, the numbers obtained with the optimal sample size (*n*=22) are closer to the expected numbers given to MultiPower than with the rest of the sample sizes.

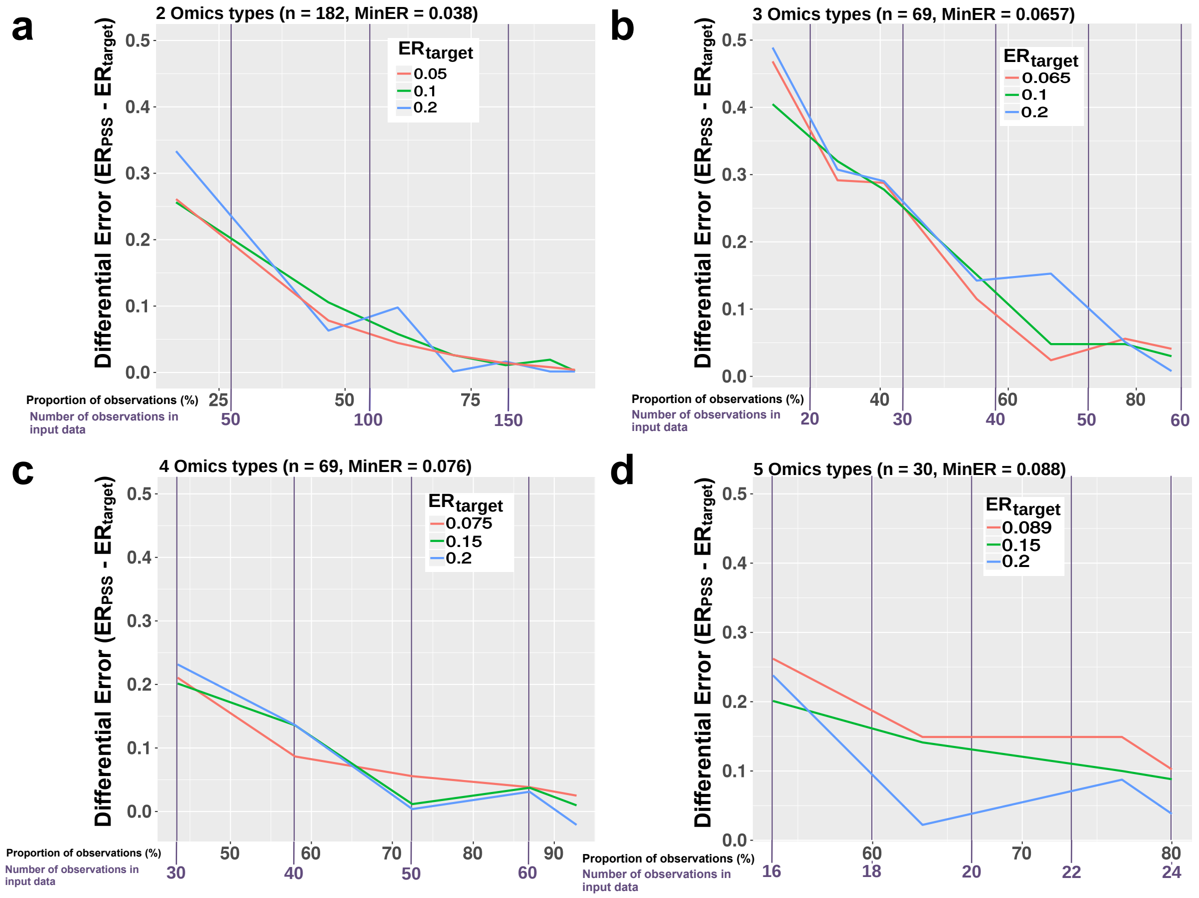

Supplementary Figure 6. MultiML use-case: TCGA gliobastoma data. Accuracy evaluation of the classification error rate models for different combinations of omics types. Each panel displays results for one omics combination. **a** Gene Expression/Proteomics. **b** Gene Expression/Proteomics/RNA-seq. **c** Gene Expression/Proteomics/RNA-seq/miRNAs/. **d** Gene Expression/Proteomics/RNA-seq/miRNAs/Methylation. The plots represent the deviation from the target error rate (ER_{target}) of the error rate achieved by the sample size predicted by MultiML (ER_{PSS}) compared with the number of samples used as input data. As expected, as the size of the input dataset increases, the accuracy of MultiML to predict a sample size that controls the target ER improves, regardless the omics combination considered. Plots also reveal that for a fixed number of observations in the input dataset, using more omics in the classifier increases the classification performance and the accuracy of MultiML. MinER: error rate achieved by the model with all samples, n: number of samples.

Being Pilot Data a list of O omics datasets O: $\{o_1, o_2, \dots, o_o\}$, with number of samples $\boldsymbol{N}$: $\{n_1, n_2, \dots, n_o\}$ and number of variables $\boldsymbol{P}$: $\{p_1, p_2, \dots, p_o\}$. Being Y the classification variable of each sample in $\boldsymbol{N}$ and being g the number of classes in Y

1: Select input parameters:

1.1: Set target error rate (ER_{target}) [Minimum ER reached in the pilot study, OR ! $\#value$ given by the user]

1.2: Set sampling strategy (SS) [Unbalanced, OR Balanced]

1.3: Set Cross-Validation method (CV) [leave-one-out OR 10-fold]

1.4: Machine Learning method (ML)[PLS-DA, OR RF, OR user-provided function]

if ML = PLS-DA *then*

set a prediction distance (PD)[maximum, OR to centroid, OR Mahalanobis]

Do

2: Create a complete multi-omics dataset $F \in O$, F: $\{o_1, o_2, \dots, o_f\}$ with vector of number of samples $\boldsymbol{N}'$: $\{n'_1, n'_2, \dots, n'_f\}$ where $n'_i \le n_i$ AND $n'_1 = n'_2 = \dots = n'_f = N_{max}$

3: Make a random selection of number of samples or ticks $\boldsymbol{\tau}$: $\{t_1, \dots, t_5\}$ applying the selected SS strategy t_i is a tick where $t_1 = 2 \text{ x } g$; $t_5 = N_{max}$

4: LASSO regression for dimensionality reduction

4.1: ∀ omics in F and ∀ tick in $\boldsymbol{\tau}$ create a subdataset S_{t_i,p_f}

4.2: ∀ S_{t_i,p_f} perform LASSO regression defining CV and ML methods to obtain $\boldsymbol{q}$ variables that best explain S_{t_i,p_f}

5: Classification methods

5.1: Use $\boldsymbol{q}$ to predict the class g in Y using ML, SS and PD methods to calculate classification error rate ER_{t_i}

5.2: Repeat 4.1 to 5.1 m times to obtain ER_{t_i} each time and calculate the average $\overline{ER_{t_i}}$ and the confidence interval $([ER_{t_i}])$

$m = 15$ as default OR it can be provided by the user

5.3: Store $\overline{ER_{t_i}}$ in vector $\overrightarrow{\mathbf{ER}_{t_i}}$ AND $[ER_{t_i}]$ in vector $\overrightarrow{[\mathbf{ER}_{t_i}]}$

6: Algorithm termination protocol

6.1:Increase vector τ with 2 more ticks t_6 and t_7 where $t_1 < t_6 < t_5$ AND $t_1 < t_7 < t_5$ AND $t_1 \neq t_2 \neq \dots \neq t_6 \neq t_7 \neq t_5$

6.2: Run steps 4 and 5 with t_6 and t_7 and store $\overline{ER_{t_6}}, \overline{ER_{t_7}}$ *in* $\overrightarrow{\mathbf{ER}_{t_i}}$ AND $[ER_{t_6}], [ER_{t_7}]$ in $\overrightarrow{[\mathbf{ER}_{t_i}]}$

6.3: *If* 3 elements of $\overrightarrow{[\mathbf{ER}_{t_i}]}$ vector overlap $\ge 12\ \%$ *then*

end

else

Back to 4.1

7: Prediction model

Fit a first-order smoothed penalized P-Spline using vector $\boldsymbol{\tau}$ vs $\overrightarrow{[\mathbf{ER}_{t_i}]}$

Use the P-Spline to calculate the sample size $N_{ER_{target}}$ for ER_{target}

END

Supplementary Figure 7. MultiML pseudocode. ER_{target}: target error rate; SS: sampling strategy; CV: cross-validation method; ML: machine learning; PLS-DA: partial least squares-discriminant analysis; RF: random forest; PD: predictor distance; $N_{ER_{target}}$: Sample size of the target error rate.

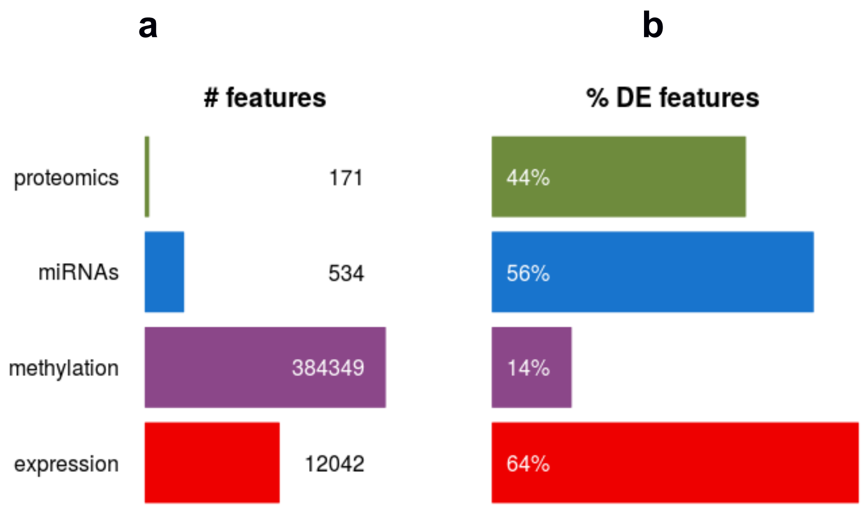

Supplementary Figure 8. TCGA glioblastoma data. Each color corresponds to a different omic modality. **a** Number of features per omic. **b** Expected percentage of differentially expressed features per omic.

Supplementary References

1 Gomez-Cabrero, D. *et al.* STATegra, a comprehensive multi-omics dataset of B-cell differentiation in mouse. *Sci Data* **6**, 256 (2019). https://doi.org/10.1038/s41597-019-0202-7

2 Verhaak, R. G. W. *et al.* Integrated genomic analysis identifies clinically relevant subtypes of glioblastoma characterized by abnormalities in PDGFRA, IDH1, EGFR, and NF1. *Cancer Cell* **17**, 98-110 (2010).

3 Ritchie, M. E. *et al.* Limma powers differential expression analyses for RNA-sequencing and microarray studies. Nucleic *Acids Res* **43**, 47 (2015).
